# *Pediludiella daitoensis* gen. et sp. nov. (Scenedesmaceae, Chlorophyceae), a large coccoid green alga isolated from a *Loxodes* ciliate

**DOI:** 10.1038/s41598-020-57423-x

**Published:** 2020-01-20

**Authors:** Ryo Hoshina, Masashi M. Hayakawa, Mayumi Kobayashi, Rina Higuchi, Toshinobu Suzaki

**Affiliations:** 1 Nagahama Institute of Bio-Science and Technology, Tamura 1266, Nagahama, Shiga 526-0829 Japan; 2 Department of Biology, Graduate School of Science, Kobe University, 1-1 Rokkodai-cho, Nada-ku, Kobe, 657-8501 Japan; 3 Present Address: Research Center for Bioinformatics & Biosciences, National Research Institute of Fisheries Science, Japan Fisheries Research & Education Agency, 2-12-4 Fukuura, Kanazawa-ku, Yokohama 236-8648 Japan

**Keywords:** Taxonomy, Symbiosis

## Abstract

Freshwater protists often harbor unicellular green algae within their cells. In ciliates, possibly because of large host cell sizes and the small size of algal coccoids, a single host cell typically contains more than a hundred algal cells. While surveying such algae-bearing protists on Minami Daito Jima Island in Japan, we found a green *Loxodes* ciliate (Loxodida, Karyorelictea) that contained one or two dozens of very large coccoid algae. We isolated one of these algae and analyzed its characteristics in detail. A small subunit (SSU) rDNA phylogeny indicated *Pseudodidymocystis* species (Scenedesmaceae, Chlorophyceae) to be the taxon closest to the alga, although it was clearly separated from this by 39 or more different sites (inclusive of gaps). SSU rRNA structure analyses indicated that these displacements included eight compensatory base changes (CBCs) and seven hemi-CBCs. We therefore concluded that this alga belongs to a separate genus, and described it as *Pediludiella daitoensis* gen. et sp. nov. The shape of the isolated and cultured *P*. *daitoensis* was nearly spherical and reached up to 30 µm in diameter. Chloroplasts were arranged peripherally and often split and elongated. Cells were often vacuolated and possessed a net-like cytoplasm that resembled a football (soccer ball) in appearance, which was reflected in the genus name.

## Introduction

Most microalgae do not have discriminative characters for the ease of taxonomy. *Scenedesmus* Meyen (Scenedesmaceae, Chlorophyceae) and its allies usually have comparatively notable morphological characters; typically, four or eight coenobia consist of ellipsoid or crescent cells, and some have spines on outermost cells (*Desmodesmus* (Chodat) An, Friedl et Hegewald).

On the other hand, coccoid green algae that are non-motile and reproduce solely by means of autosporulation are one of the most difficult group for taxonomy. They had been classified to a single group Chlorococcales^1^. However, due to the development of molecular phylogenetic analysis, it has been shown that such coccoid algae sporadically distribute into both Chlorophyceae and Trebouxiophyceae^2,3^. Family Scenedesmaceae has no exception to this rule. The coccoid genera, such as *Hylodesmus* Eliáš, Němcová, Škaloud et Neustupa^4^, *Graesiella* Kalina et Puncochárová^5^, and many unidentified coccoids^6,7^ are sporadic in the Scenedesmaceae. This also includes some species that had belonged to the genus *Chlorella* Beijerinck in previous times. For example, *Chlorella fusca* Shihira et Krauss was once a species with several varietas, but their polyphyletic status showed that some of them were synonyms of *Graesiella* species^5^. *Paramecium bursaria*, a model organism of algae-bearing ciliates that establish stable symbioses, generally possesses *Chlorella* or its allies as symbionts^8^. However, in some particular strains, it has been found to possess algae belonging to Scenedesmaceae^7^.

In freshwater environments, many protozoa generally harbor numerous green coccoids within a single cell. Ciliates (Alveolata) are the most common among such protozoans, along with certain Amoebozoa and Heliozoa^9^. These protists typically contain hundreds of algae within a single host cell, and we referred to them collectively as multi-algae retaining protists (MARP)^10^. In the course of our investigation of MARP on a subtropical island in Japan, we found a green *Loxodes* ciliate (Loxodida, Karyorelictea) that contained between one and two dozens of very large coccoid algae. We isolated this alga and found that this alga occupies a peculiar position in the SSU rDNA tree in the Scenedesmaceae. To learn more about this alga, we executed detailed morphological and ultrastructural observations and molecular analyses including SSU and ITS secondary structure comparisons as well as SSU rDNA phylogeny. We conclusively describe a new genus and species, *Pediludiella daitoensis* gen. et sp. nov. (Scenedesmaceae, Chlorophyceae).

## Results

### Ciliate observation and algal isolation

**Figure 1:**
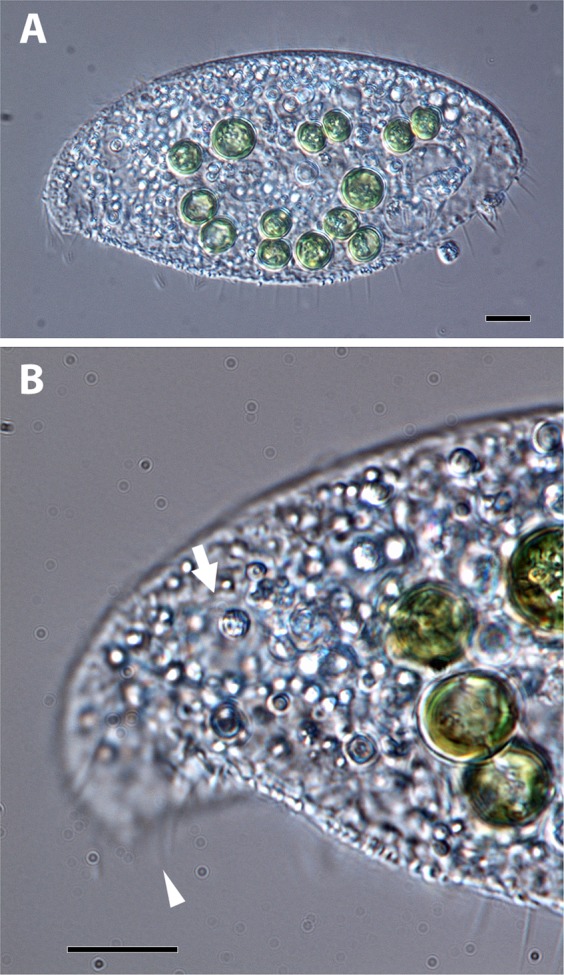
Light micrographs of a living cell of *Loxodes* sp. (**A**) A whole cell containing significantly large green-colored algae. (**B**) Enlarged micrograph of the anterior part of the same cell in A, showing a strongly bent apex (arrowhead) and a Müller vesicle (arrow), which are key features of the genus *Loxodes*. Scale bars: 10 µm.

Water samples, including leaf debris, were collected from a freshwater pond on Minami Daito Jima (South Borodino Island), Okinawa, Japan, and were cultured for approximately 10 days with wheat grains as a nutrient source. Three morphotypes of small animals or protozoa harboring green coccoids subsequently emerged, one of which was a green *Hydra* (Cnidaria). The other two were ciliates, of which one was a species of *Frontonia* (Peniculida, Oligohymenophorea), and the other was a species belonging to the family Loxodidae (Karyorelictea) because it contained Müller vesicles (Fig. 1, Suppl. Video 1). This Loxodidae ciliate was further identified as *Loxodes* sp., since it was a freshwater species characterized by an elongated oval and flat body with a strongly curved anterior end^11^. The algae within *Hydra* and *Frontonia* resembled the so-called *Chlorella*-like algae (ca. 4–5 µm in size) and hundreds of these cells were observed in each host individual (data not shown). In contrast, the algae in *Loxodes* were significantly larger in size than those in the other two host species (more than 10 µm in diameter) and smaller in number (between one and two dozens) (Fig. 1). We isolated this alga and established an algal strain (LxSd1).

### Light microscopy

**Figure 2:**
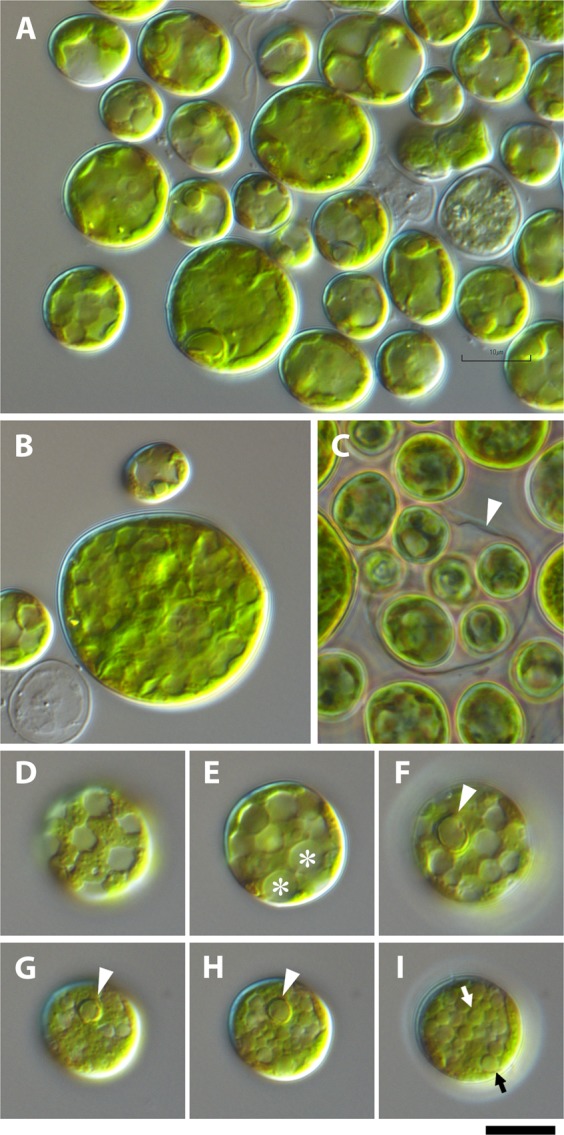
Light micrographs of cultured LxSd1 cells taken with differential interference (**A**,**B**,**D**–**F**) or phase contrast optics (**C**). (**A**) Coccoids of various sizes. (**B**) A large cell with a diameter of 25 µm or more. (**C**) Several daughter cells hatching from a mother cell. Phase contrast microscopy clearly shows the cell wall of the mother cell (arrowhead). (**D**–**F**) Micrographs of the same cell (possibly at the early stationary stage) taken at different focus points. The reticulated green-colored cytoplasm is clearly shown. The small round structures (asterisks) show a differential interference effect that contrasts with that of the cell body, indicating that these are vacuoles containing fluid with a density lower than that of the cytoplasm. One large high-density granule of 2–3 μm in diameter was observed in the cell that showed the same differential interference effect as the cell body (arrowhead in (**F**)), which appeared to be a pyrenoid. (**G**–**I**) Micrographs of the same cell (possibly at the late stationary stage) taken at different focus points. One pyrenoid-like granule was also found in this cell (arrowheads in (**G**,**H**)), and many smaller granules of high optical density were also observed (arrows in (**I**)). Scale bar: 10 µm.

Cells of the isolated and cultured LxSd1 were nearly spherical and between 7.2 and 30 µm in diameter (Fig. 2). Distinct single pyrenoids were often observed in individual cells (arrowheads in Fig. 2F–H). Cells were often vacuolated (Fig. 2D–F), and/or sometimes contained small granules with a higher refractive index than that of the cytoplasm (arrows in Fig. 2I). Because of this, the cytoplasm containing chloroplasts sometimes resembled a net (Fig. 2D–F). The cells were observed to divide by autosporulation, forming 4 to 16 daughter cells from a single mother cell (Fig. 2C); however, no coenobia were observed. LxSd1 showed a certain degree of aggregation (Fig. 2A), and shaking culture induced the formation of distinct cell masses (data not shown) without noticeable gelatinous material.

**Figure 3:**
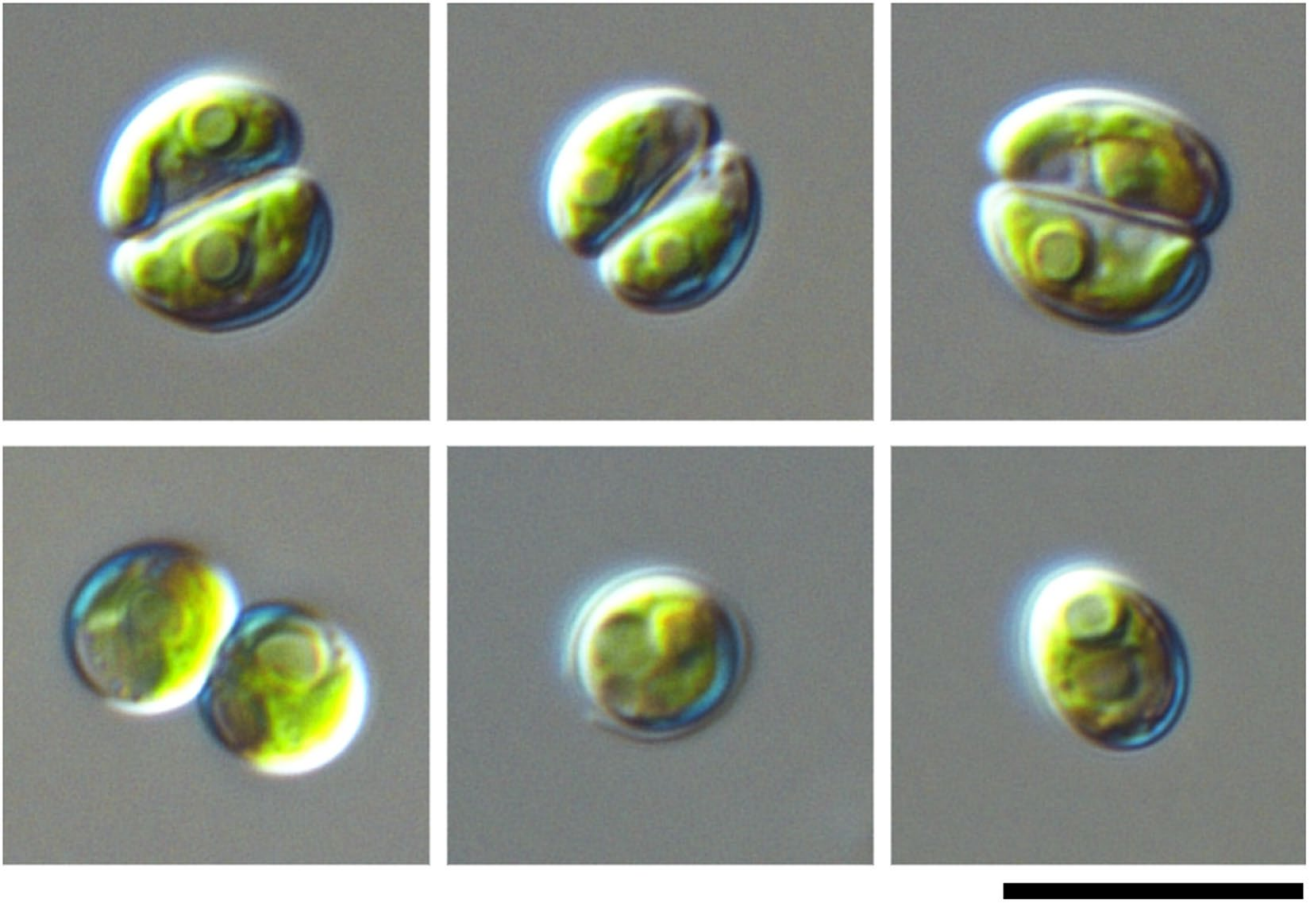
Light micrographs of *Pseudodidymocystis* species. Upper pictures are of *P*. *planctonica* SAG 40.98. Bicellular coenobia. Lower pictures are of *P*. *fina* SAG 2088. *Pseudodidymocystis planctonica* almost always formed bicellular coenobia, whereas *P*. *fina* formed both bicellular coenobia and single cells. Scale bar: 10 µm.

Microscopic examination of the closely related algae (see SSU rDNA phylogeny shown below), *Pseudodidymocystis planctonica* (Korshikov) Hegewald & Deason SAG 40.98 and *P*. *fina* (Komárek) Hegewald & Deason SAG 2088 was also performed for comparison (Fig. 3). Unlike LxSd1, bicellular coenobia with long and bent cells constituted most of the *P*. *planctonica* culture. In contrast, ellipsoidal or ovoid single cells accounted for the majority of the *P*. *fina* stock, and bicellular coenobia were in the minority, which also differs from LxSd1.

### Transmission electron microscopy

**Figure 4:**
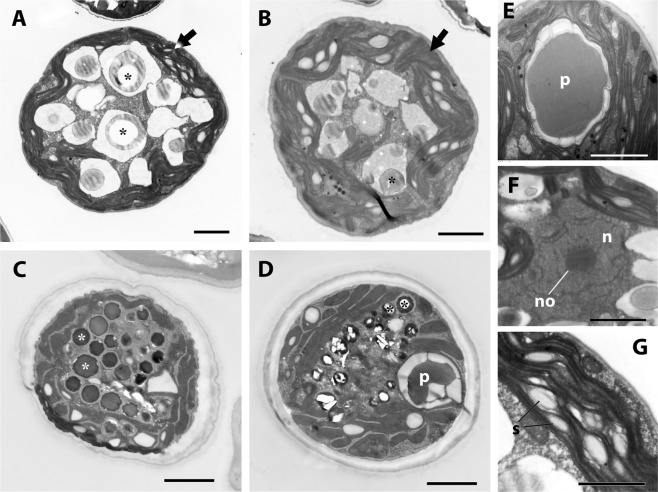
Transmission electron micrographs of LxSd1. (**A**,**B**) Typical cells at the early stationary stage. Large vacuoles of 2–3 μm diameter, containing spherical inclusions (asterisks) can be seen. These inclusions often had hollow portions. The cytoplasm was mainly occupied by chloroplasts (arrows). (**C**,**D**) Cells at the late stationary stage (near starvation). Many electron-dense granules (asterisks) are seen, which appeared to be surrounded by a membrane. (**E**) A pyrenoid (p) surrounded by segmented starch granules. Split and elongated chloroplasts also can be seen. (**F**) Nucleus (n) with a centrally located single nucleolus (no). (**G**) Starch granules (s) are located between thylakoid membranes in the chloroplast. Scale bars: A–F, 2 µm; G, 1 µm.

Cells in the early stationary phase typically contained several large vacuoles, in which some granular structures were observed (Fig. 4A,B). The cytoplasm contained chloroplasts surrounding the vacuoles. Small starch granules were seen between the thylakoid membranes (Fig. 4G), whereas we did not detect any of the larger granules that are generally observed in many chlorophytes. Chloroplasts were basically arranged peripherally (Fig. 4A–C), whereas split and elongated chloroplasts were often seen (Fig. 4D,E). Each cell contained a single pyrenoid, which was surrounded by segmented starch granules. (Fig. 4D,E). We did not observe any evidence of penetration of the thylakoid membranes into the pyrenoid. Although no large vacuoles were observed in the late stationary phase (cells in the near-starved state), we did detect vesicles containing granules with high electron density (Fig. 4C,D).

### rDNA sequence and a search for the most related taxa

A sequence of 2,724 bases, including small subunit-internal transcribed spacer (SSU-ITS) rDNA (SSU, ITS1, 5.8 S, ITS2, 5′ LSU rDNA), was obtained for LxSd1 (LC477067). No group I introns were found in this sequence. We conducted a search for sequences matching the LxSd1 SSU rDNA sequence using the Basic Local Alignment Search Tool for Nucleotides [BLASTN, National Center for Biotechnology Information (NCBI)]. The best matched sequences were those of scenedesmacean genera (Sphaeropleales, Chlorophyceae), including *Pectinodesmus* Hegewald, Wolf, Keller, Friedl et Krienitz, *Acutodesmus* (Hegewald) Tsarenko, and *Scenedesmus* Meyen; however, in all cases, the identities did not exceed 97%.

SSU rDNA sequences for Scenedesmaceae were obtained by searching the keywords of [scenedesmaceae + ssu] from the NCBI database, from which we removed group I introns and roughly aligned them, and subsequently constructed a neighbor-joining (NJ) tree. On the basis of resulting tree, we found LxSd1 to be most closely related to *Pseudodidymocystis planctonica* (AB037087). It appeared that several introns intervening in its SSU rDNA prevented us from performing a successful BLASTN search. According to AlgaeBase^12^, four species names are flagged as currently accepted taxonomically in the genus *Pseudodidymocystis* Hegewald et Deason: *P*. *planctonica* (type species), *P*. *fina*, *P*. *inconspicua* (Korshikov) Hindák, and *P*. *lineata* (Korshikov) Hindák. *Pseudodidymocystis planctonica* and *P*. *fina* were found to be held in the Culture Collection of Algae at the University of Göttingen (SAG); however, we were unable to locate either of the other two species in any of the searched culture collections. The published sequence of *P*. *planctonica* (AB037087) contains several Ns, indicating uncertain positions, and comprehends only SSU rDNA. We thus aimed to improve the quality and length of the sequence. Accordingly, we obtained samples of the former two species, *P*. *planctonica* SAG 40.98 and *P*. *fina* SAG 2088, and sequenced these. The rDNA sequences of 5,052 bases for *P*. *planctonica* (LC477068) and 5,416 bases for *P*. *fina* (LC477069) were thus obtained. These sequences included numerous introns. For *P*. *planctonica*, the SSU rDNA contains five group I introns and the LSU rDNA contains a single intron, whereas in *P*. *fina* these rDNAs contain six introns and one intron, respectively (Suppl. Fig. 2). Reference to the Comparative RNA Web Site^13^ [http://www.rna.ccbb.utexas.edu/] indicated that six group I introns intervening in SSU rDNA will be the highest number ever recorded in the Viridiplantae alongside *Selenastrum capricornutum* Printz (AF169628, Chlorophyceae).

### SSU rDNA phylogeny

**Figure 5:**
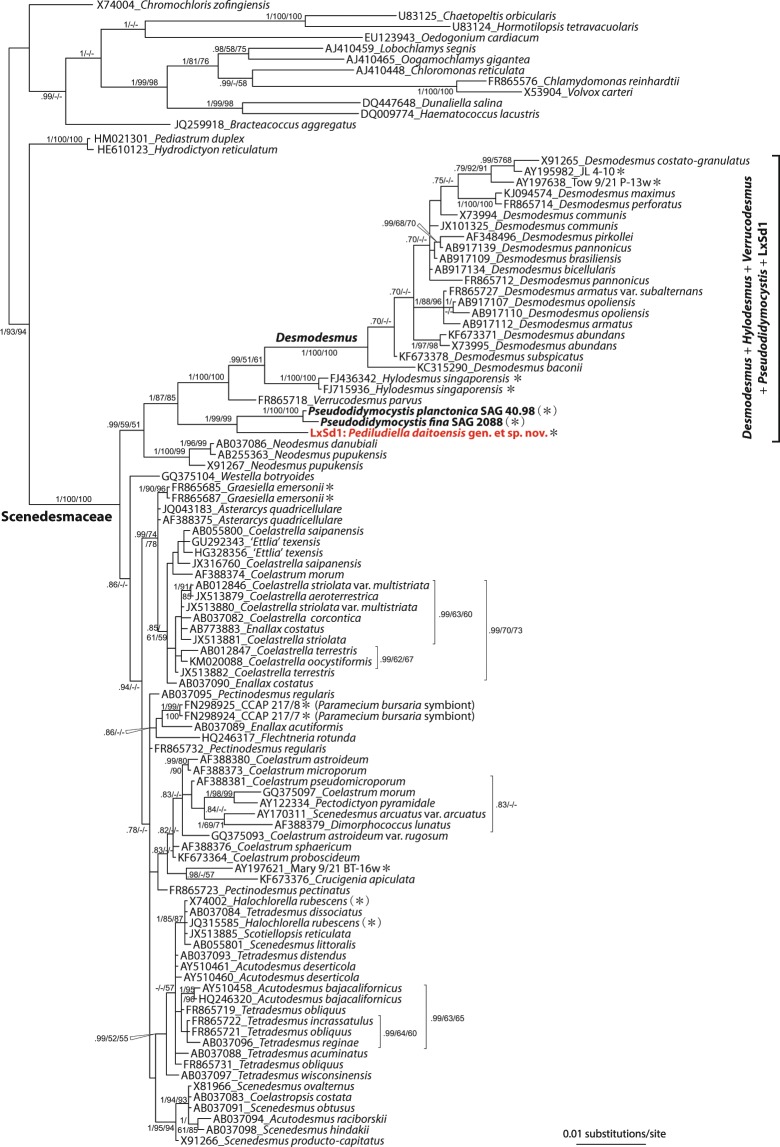
Bayesian inference tree for Scenedesmaceae based on SSU rDNA sequences. Numbers at the main branches correspond to MrBayes posterior probabilities/maximum likelihood/neighbor-joining bootstrap values. Hyphens correspond to Bayesian posterior probability values below 0.70 and bootstrap values below 50%. Algal strains sequenced in this study are shown in bold type. The scenedesmacean species with a spherical shape are indicated with asterisks, and species occasionally spherical shape are indicated with asterisks in parentheses.

Scenedesmacean SSU rDNA sequences collected above were aligned with those of several outgroup taxa. Phylogenetic trees were constructed using Bayesian inference (BI), maximum likelihood (ML) and NJ methods, and we here show the resultant BI tree, together with Bayesian posterior probabilities (PP) and the bootstrap values (BVs) of both ML and NJ analyses. The tree clearly shows the monophyly of the family Scenedesmaceae (Fig. 5). Although, branching of more internal nodes is not clear, several clades can be regarded. *Desmodesmus*, a genus characterized by coenobia, of which the outer-most cells have bent spines, was clearly separated from other genera. The monophyletic relationship of *Desmodesmus*, *Hylodesmus* Eliáš, Němcová, Škaloud, Neustupa, Kaufnerová et Šejnohová and *Verrucodesmus* Hegewald was perfectly supported in all analyses (Suppl. Figs. 3 and 4). LxSd1 was clustered with *Pseudodidymocystis* species, and this group was placed as a sister group to *Desmodesmus* + *Hylodesmus* + *Verrucodesmus* clade. Monophyly of all the above taxa (i.e., *Desmodesmus* + *Hylodesmus* + *Verrucodesmus* + *Pseudodidymocystis* + LxSd1; DHVPL clade) was relatively highly supported by PP = 1 and more than 80% BVs. *Neodesmus* Hindák species lied sister to DHVPL clade. Although this branch was not highly supported by 0.99, 59 and 51 (PP, MLBV, NJBV), it has also been found in other recent studies^4,14^. Coccoid scenedesmids are occasionally found in DHVPL clade, as well as in the other scenedesmacean linages (asterisks in Fig. 5), and which has been verified in other studies^4,15^.

### SSU rRNA structure

**Figure 6:**
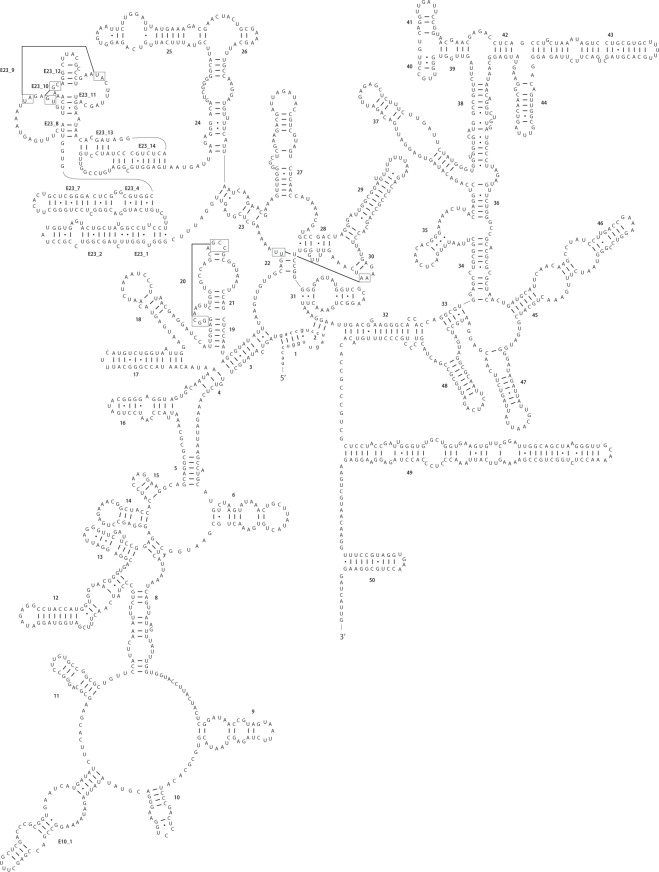
Predicted SSU rRNA secondary structure for LxSd1 (LC477067). The molecule is roughly oriented as a clockwise circle in 5′–3′orientation. Each helix is labelled according to eukaryotic model by Wuyts *et al*.^27^. The 20 characters in lower-case letters at the 5′ terminus constitute the primer-binding region.

**Figure 7:**
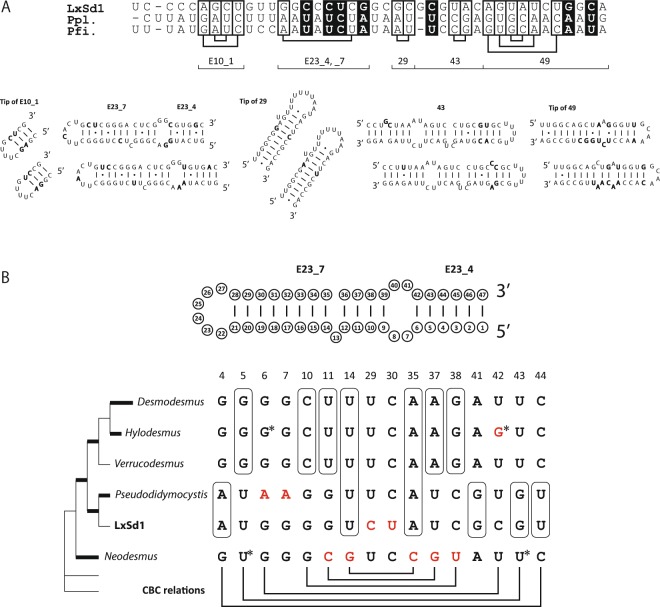
Variable sites in SSU rRNA. (**A**) Variable sites among LxSd1 and *Pseudodidymocystis* species (Ppl: *P*. *planctonica*, Pfi: *P*. *fina*), and these positions in the secondary structure. Compensatory base changes (CBCs) detected between LxSd1 and *Pseudodidymocystis* species are boxed and connected, whereas hemi-CBCs are marked by a black box. (**B**) Comparison of SSU rRNA helix E23_4 to 7 structure among *Desmodesmus*, *Hylodesmus*, *Verrucodesmus*, *Pseudodidymocystis*, LxSd1 and *Neodesmus*. Base changes characterizing genera or clades are shown. Synapomorphic changes supporting the clade monophylies are circled. The non-homoplasious synapomorphies are in red, and CBC relations are shown. Changes of base no. 29 and 30 are hemi-CBCs. Asterisks indicate uncoupled substitutions. For better understanding, a simplified tree (see, Fig. 5) is provided. Branches significantly supported by Bayesian posterior probabilities >0.95 and bootstrap values >80% are shown in bold.

We detected 39 sites (including gaps) that differ between the SSU rDNA sequences of LxSd1 and *Pseudodidymocystis planctonica*, and 40 sites that differ between LxSd1 and *P*. *fina*. We constructed a secondary structure model for LxSd1 SSU rRNA (Fig. 6) and made accurate comparisons with models for *Pseudodidymocystis* species (Fig. 7A). Base displacements between LxSd1 and *Pseudodidymocystis* were somewhat concentrated in specific helices; for example, eight substitutions were detected in helix E23_4–7, six in helix 43, and 11 in helix 49. These displacements included compensatory base changes (CBCs, mentioned later). In addition, we detected five base displacements among *Pseudodidymocystis* species, all of which are located in single-stranded sections, such as loops or bulges (data not shown).

### ITS structure

**Figure 8:**
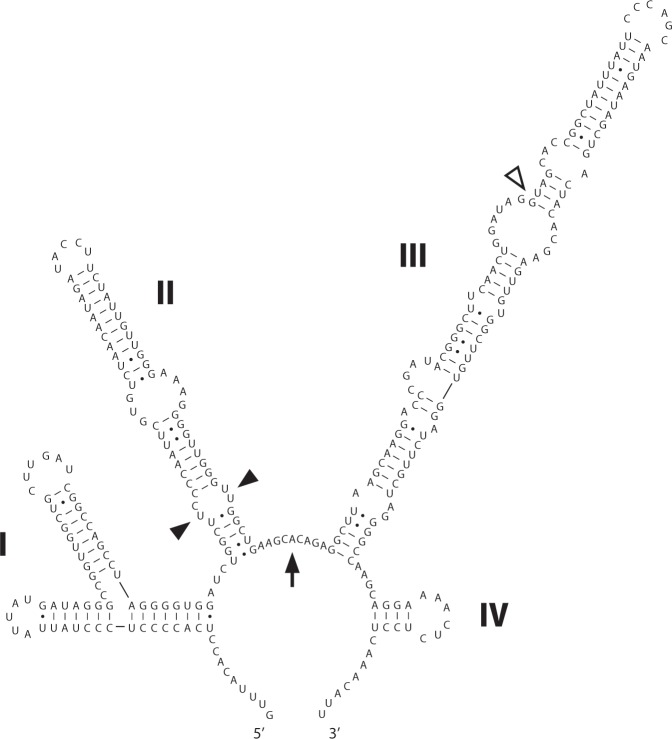
Predicted ITS2 secondary structure diagrams for LxSd1. Roman numerals (I to IV) indicate the helix number. The figure shows the typical structural motifs for eukaryotes, namely, the pyrimidine–pyrimidine mismatch (indicated by arrow heads) in helix II, an A-rich region (arrow) between helices II and III, and the GGU motif (outlined arrow head) on the 5′ side of the apex of helix III. A branched helix I is typical of Sphaeropleales.

Currently, the primary and secondary structures of ITS2 are considered to be important in terms of “species” determinations. The predicted secondary structure of LxSd1 ITS2 is shown in Fig. 8. Well-known sequence motifs, including a U-U mismatch in helix II and an A-rich region between helices II and III, as well as a GGU triplet on the 5′ side of helix III, are present. A y-shaped helix I is typical of Sphaeropleales^16–18^. Helices II and III of LxSd1 ITS2 are elongated when compared with those of *Pseudodidymocystis* species (data not shown). When gap sites were considered as different sites, the differences between LxSd1 ITS2 and the ITS2 of both *Pseudodidymocystis* species reached 35.5%. Even if gap sites were not counted, the differences reached 23%.

ITS1 structures for LxSd1 (Suppl. Fig. 5) and *Pseudodidymocystis* species (data not shown) were build. Four common helices were found among them. The different sites between LxSd1 and *Pseudodidymocystis* species for these helix regions reached 26% (gap sites were not counted) or 35% (gap sites were considered as different sites). For LxSd1, an additional helix was predicted between the third and fourth helices (Suppl. Fig. 5), that was not found in *Pseudodidymocystis* species.

## Discussion

Most microorganisms are difficult to culture^19,20^. If there is a protozoan with algae in the cell, it is difficult to determine if it is truly symbiotic or not without long-term culture. However, regardless of whether it was true symbiosis (i.e., long-time retention) or not (temporally retention), ciliate-algae combinations in natural water environment are usually observed as hundreds of small coccoid algae per one ciliate cell^9,10,21^. In this study, we focused on a *Loxodes* that retains between a dozen and two dozens of very large coccoid algae (Fig. 1). *Loxodes* is known as a ciliate that is difficult to culture. We could not maintain this green *Loxodes*, therefore, we could not verify whether it was a true symbiosis or not. However, for several days between its appearance and disappearance, we did not observe the algae inside of *Loxodes* looked during digestion. We, therefore, considered these large coccoids as more likely to have the endosymbiotic nature, and there is a possibility that the *Loxodes* live under mixotrophic nutrition. In fact, a *Loxodes* strain with algal symbionts has been reported^22^.

Our phylogenetic analyses indicated that LxSd1 undoubtedly belongs to the Scenedesmaceae, in which formed a robust relationship to *Desmodesmus*, *Hylodesmus*, *Verrucodesmus* and *Pseudodidymocysti* (Fig. 5). The LxSd1 constructed a sister relationship with *Pseudodidymocystis*. Cyclic morphological change, i.e., single cell/two-celled coenobia, which is dependent on culture conditions, is one of the defining characters of *Pseudodidymocystis* species^23^. Under our culture conditions, *P*. *planctonica* almost always formed bicellular coenobia, whereas *P*. *fina* formed both bicellular coenobia and single cells (Fig. 3). The latter species sometimes formed nearly spherical cells (Fig. 3). Table 1 shows morphological outlines of coccoid genus *Hylodesmus* and *Pseudodidymocystis* species. *Pseudodidymocystis* species usually exhibit bicellular coenobia by elongated oval or ellipsoidal cells, when they exhibit nearly spherical, diameter close to the minor axis of ellipsoids^23–25^. That is, the diameter will be around 3 to 5 µm. *Pseudodidymocystis* species are also characterized by having a particular cell wall consisting of a fibrous inner layer and a sporopollenin outer layer that often granulated^23^. Such particular cell wall structures were not observed in LxSd1 cells (Figs. 2 and 4). *Hylodesmus* reaches 11 µm in diameter. This species was also characterized by having a particular cell wall composed of a thick inner layer and a thin trilaminar outer layer, whose surface was ornamented with a few ribs^4^. In the cells of LxSd1, we found several large vacuoles containing a few granular structures in early stationary phase (Fig. 4A,B). And also, we found many vesicles containing granules with high electron density in the late stationary phase (Fig. 4C,D). These granules may be derived from the low electron density granules that appeared in the early stationary phase, and are considered to be a unique nutrient store for LxSd1. Numerous high electron density vacuoles were also found in *Hylodesmus*, but not found nested constructions^4^.

**Table 1:** Morphological outlines of LxSd1, *Pseudodidymocystis* species, and *Hylodesmus singaporensis*.

	Cell size* (µm)	Autospore	Chloroplast	Outer ornament^†^	References
LxSd1	φ 7–30	4–16	Split lobes or net-like	none	This study
*P*. *planctonica*	4.2–8.8 × 2.5–4.2	2	Discoid	none	^23^
*P*. *fina*	4.5–7.5 × 2.5–3.6	2	n.d.	none	^23^
*P*. *inconspicua*	5–9.5 × 1.6–4	4	Parietal	Longitudinal ribs	^25^
*P*. *lineata*	4.8–9.2 × 3.5–4.4^‡^	2	n.d.	Longitudinal ribs	^24,25^
*H*. *singaporensis*	φ 4.2–11	2–16	Girdle	Ribs	^4^

*In one instance of culture. ^†^Only stand out structures. Besides, *Pseudodidymocystis* is characterized by having a sporopollenin cell wall layer. Cell walls usually are smooth, but often granulated and/or covered with mucilage. ^‡^Measured from the figure of Hindák^25^. n.d., not determined.

In terms of the morphology of LxSd1, the large cell size, and split chloroplasts are particularly prominent features of this alga (Figs. 2 and 4). Coccoid green algae have often been lumped together as *Chlorella*^6^, but the features of LxSd1 differ from such ‘*Chlorella*-like’ species. Several coccoid algae with split chloroplasts have been known, but most of them belong to remote lineages, Chlamidomonadales (Chlorophyceae) or Trebouxiophyceae. Among the scenedesmacean taxa, *Graesiella emersonii* (Shihira et Krauss) Nozaki Katagiri, Nakagawa, Aizawa et Watanabe would be the most resembling species in this respect, but it is located outside the clade that we are focusing on (Fig. 5). *Graesiella emersonii* grows up to 17 µm in diameter and equipped slit chloroplast^5^. However, *G*. *emersonii* exhibits multinuclearity in mature cells, which was never found in LxSd1.

To the best of our knowledge, the secondary structure model for the chlorophycean SSU rRNA of *Volvox carteri* Stein published in 1989^26^ is still the most recent available model of this molecule. However, in the portion between helices 18 and 19 of this model (these fall under the helices 23 and 24 in Fig. 6), the pairing states of over 200 nucleotides were not indicated. Coincidentally, in our comparisons of SSU rDNA between LxSd1 and the most closely related *Pseudodidymocystis* species, nearly 20% of the detected differences were concentrated in this portion. Recent structural studies in other organisms have presented revised structural models^27–29^, and therefore, in the present study, we present a new structural model for Chlorophyceae, thereby filling the existing gap. As a consequence, we reveal the presence of helix E23, including 11 small helices (Fig. 6), which was not shown in the *Volvox* model. In our comparison between LxSd1 and *Pseudodidymocystis* species, we detected differences in eight CBCs and seven hemi-CBCs, among which one and four, respectively, were located in helix E23 (Fig. 7A). A CBC occurs when one side of a base pair changes followed by a change on the opposite side, conserving the site as a complementary base pair in a double-stranded helix. If only one side changes, the resulting structure is referred to as a hemi-CBC. Both CBCs and hemi-CBCs are used to establish the confidential folding pattern of rRNAs^30^. In contrast, we detected no substitutions in pairing regions between the *Pseudodidymocystis* species and found only a few base changes and indels that occurred only in loops or bulges. (Fig. 7A). These results support not only the accuracy of the structural model, including helix E23, but also the high differentiation between LxSd1 and *Pseudodidymocystis* species and the higher conservation of the helices among *Pseudodidymocystis* species.

It has been regarded that multiphasic approach, such as the combination of morphology, ultrastructure, physiology, phylogeny and reproductive isolation, is ideal for algal taxonomy^31–33^. However, it has been known that morphological or physiological characters can vary according to the environmental conditions^2,29^. In the case of members of the Chlorellaceae (Trebouxiophyceae, asexual reproducing by autospores), for which only a small number of morphological characters can be used to separate genera, it has been initiated with the introduction of only the differences of rDNA sequences with no recourse to multiphasic approach. Now, many genera in the Chlorellaceae are defined by the molecular phylogeny, CBC and synapomorphic base change or indels in SSU or ITS2 rDNA found in specific clades^34^. Looking at each clade (Fig. 5) from this perspective, it can be characterized by many CBCs and non-homoplasious synapomorphies (NHS, according to Marin *et al*.^28^). Here we show the base comparison data at helix E23_4 to 7 as a representative (Fig. 7B). Some synapomorphic CBC supported the monophylies of *Pseudodidymocystis* + LxSd1 clade as well as *Desmodesmus* + *Hylodesmus* + *Verrucodesmus* clade. The NHS were found in *Hylodesmus*, *Pseudodidymocystis*, and *Neodesmus*. Changes of base no. 29 and 30 are unique hemi-CBCs, not found in any other scenedesmids used in the phylogenetic analyses (all members had U and C, data not shown), therefore, we concluded that these changes were equivalent to NHS (Fig. 7B).

Although it has been argued that the presence of a CBC in the ITS2 between two organisms constitutes strong statistical evidence to separate them as different species^35–37^, there are no statistical data concerning generic or species separation based on CBCs in SSU rRNA. In a case of the Chlorellaceae, the genus *Carolibrandtia* Hoshina et Nakada (including only one species) was established on the basis of a single unique CBC in the SSU rRNA, which supported its phylogenetic separation from other genera^38^. In this case, the number of substitutions against the closest species of other genera was only eleven (*Car*. *ciliaticola* (Hoshina *et al*.) Hoshina et Nakada [LC228604] vs. *Chlorella sorokiniana* Shihira et Krauss [AB731602]). In one of the latest paper on the chlorophycean taxonomy, Liu *et al*.^39^ recognized a phylogenetically separated group among filamentous algal family Chaetophoraceae, and elected a new genus *Chaetophoropsis* B. Wen Liu *et al*. Within this genus, the maximum difference between SSU rDNA sequences reaches 13 sites (*Cha*. *polyrhizum* (Jao) B. Wen Liu *et al*. [MF497328] vs. *Cha*. *pisiformis* var. *hamata* (Jao) B. Wen Liu *et al*. [MH002612]), whereas, the minimum difference between those of new genus and existing genera remained at 14 (*Cha*. *elegans* (Roth) B. Wen Liu *et al*. [FN824388] vs. *Fritschiella tuberosa* Iyengar [FN824385]). In the present study, therefore, the difference in 39 or more sites, including eight CBCs and seven hemi-CBCs, between LxSd1 and *Pseudodidymocystis* species could be a justification for genus separation.

Accordingly, on the basis of the observations made in this study, we conclude that the algal strain LxSd1 is a new species belonging to a new genus. We therefore propose the name *Pediludiella daitoensis* gen. et sp. nov. ITS2 data (Fig. 8) will be useful for delimiting the species when related species are discovered.

## Description

*Pediludiella* Hoshina, Hayakawa et Suzaki gen. nov. (Scenedesmaceae, Chlorophyceae).

Diagnosis: Vegetative cells solitary, spherical or ellipsoidal, or ovoid, uninucleate. Chloroplast parietal, split lobes or net-like, containing small starch grains between the thylakoid membranes. Single pyrenoid covered with starch grains. Cells often contain some vacuoles or many granular-like structures. Asexual reproduction by autosporulation, sexual reproduction not known. Genus differing from other genera of the family with regards to the nucleotide sequence and secondary structure of the nuclear SSU rRNA.

Typus generis: *Pediludiella daitoensis* Hoshina, Hayakawa, Kobayashi, Higuchi et Suzaki.

Etymology: the genus name *Pediludiella* is derived from the Latin term *pediludium* (=soccer or association football). The name is based on the fact that some cells with a net-like cytoplasm (Fig. 2D–F) invoke the image of a truncated icosahedron, comparable to the typical design of soccer balls that appeared around 1970 (black and white “Telstar”; Adidas, Germany).

*Pediludiella daitoensis* Hoshina, Hayakawa, Kobayashi, Higuchi et Suzaki sp. nov.

Figures 2 and 4.

Diagnosis: Vegetative cells solitary, spherical or ellipsoidal, or ovoid, 7–30 µm in diameter. Uninucleate. Chloroplast parietal, split lobes or net-like, containing small starch grains between the thylakoid membranes. Single pyrenoid covered by segmented starch. Cells often contain some vacuoles or many granular-like structures. Asexual reproduction by four, eight, or 16 autospores. Known only from *Loxodes* sp. (Ciliate). Whether the alga exists free-living in natural aquatic environments is not known.

Holotype: NIES-50016 strain permanently cryopreserved (metabolically inactive state) in the Microbial Culture Collection at the National Institution for Environmental Studies (NIES), Tsukuba, Japan.

Authentic culture: NIES-4031.

Type locality: Endocyte of *Loxodes* ciliate collected from Gonzoike Pond (25.842N, 131.231E) on South Borodino Island (Minami Daito Jima), Japan.

Etymology: The specific name *daitoensis* refers to the Island on which the type material was collected.

## Methods

### Crude culturing and algal isolation

Water containing dead leaves, twigs, or the remnants of submerged plants was sampled from a pond (25.842N, 131.231E) on Minami Daito Island, Okinawa, Japan, on December 5, 2010. The water sample was brought back to the laboratory at Kobe and was crudely cultured with a few grains of wheat in Petri dishes. A few days later, a *Loxodes* ciliate containing green coccoids within their bodies was observed. An individual of *Loxodes* was isolated using an elongated Pasteur pipette under a stereoscopic microscope and transferred into a depression slide filled with natural drinking water. The *Loxodes* specimen was washed through the tip of a micropipette and transferred into another depression, with this process being repeated twice. Thereafter, the isolated *Loxodes* was placed onto a 1% agar plate containing C medium^40^. Under these conditions, the *Loxodes* cell began to rupture and some of the dispersed algae survived to form colonies. The plate was incubated at room temperature for 6 weeks under illumination with LED light (14 h/10 h light/dark cycle). One small single colony (ca. 300 µm) that appeared was transferred to liquid C medium. After culturing for some time, the culture was treated with antibiotics (ampicillin 200 µg/mL + tetracycline 25 µg/mL final concentrations) for a few days, and thereafter diluted and spread onto a 1% agar plate containing 1/5 G medium^38^. From among the resulting colonies, a small single colony was picked up and transferred to liquid 1/5 G medium. The algae finally obtained in this culture formed large coccoid cells that closely resembled those observed in *Loxodes* cells. Therefore, we presumed that this alga was almost certainly derived from *Loxodes* and named it LxSd1.

*Pseudodidymocystis planctonica* SAG (Culture Collection of Algae at the University of Göttingen Germany) 40.98 and *P*. *fina* SAG 2088 obtained from SAG were also cultured under the same conditions as LxSd1.

### Cytological observations

For transmission electron microscopy (TEM), cells of LxSd1 were chemically fixed with glutaraldehyde and osmium tetroxide according to the method of Song *et al*.^41^. The fixed samples were dehydrated through a graded ethanol series and embedded in Spurr’s resin. Ultrathin sections were stained with EM stainer (Nisshin EM, Tokyo) and lead citrate before being observed by TEM (Hitachi H7100).

### DNA extraction, amplification, and sequencing

Extraction of DNA from LxSd1 and the *Pseudodidymocystis* species was performed using a NucleSpin Plant II kit (Macherey-Nagel, Düren, Germany) with modified cell fracturing. Fifty milligrams of cells were homogenized in 500 µL of preheated (65 °C) lysis buffer PL1 containing 10 µL of RNase A. After the addition of 400 µL of glass beads (ø 0.1 mm), each sample was smashed using a BeadSmash 12 device (WakenBTech, Kyoto, Japan) at 5,000 rpm for 30 s. This procedure was repeated five times, and then each sample was incubated for 10 min at 65 °C. Subsequent procedures were performed in accordance with the manufacturer’s instructions.

PCR was performed to amplify the SSU to ITS (internal transcribed spacer) rDNA region using KOD FX NEO (Toyobo, Osaka, Japan) using the primer pairs SR-1^42^ (5′ SSU)/SR-12k^43^ (3′ SSU) and INT-5F (3′ SSU; —TTC ATT AAA CCC TCC CAC CT—)/HLR3R^43^ (5′ LSU). The PCR conditions were as follows. An initial denaturation step of 94 °C for 2 min was followed by 45 cycles of the following condition: 10 s at 98 °C, 30 s at 52 °C and 90 s at 68 °C. The amplification was finished with a final step of 68 °C for 1 min. PCR products were checked once by agarose gel electrophoresis, then purified using a NucleoSpin Gel and PCR Clean-up kit (Macherey-Nagel), and finally directly sequenced using an ABI PRISM 3100 Genetic Analyzer (Applied Biosystems, Foster City, CA), using BigDye Terminator chemistry (Applied Biosystems).

### Phylogenetic analyses

SSU rDNA sequences for the scenedesmacean species were obtained by searching the keywords of [scenedesmaceae + ssu] from the NCBI database. Having been roughly aligned via Clustal X2^44^, the shorter sequences and sequences including several ‘N’ were removed. The sequences of most unidentified species not directly related to LxSd1 were also removed. Thereafter, multiple sequences from the same species were narrowed down to one representative sequence. Inserted group I introns were removed following the method of Hoshina^45^. These sequences were then aligned with several outgroup taxa considering the above secondary structures (Fig. 6).

A bootstrap tree was constructed using the neighbor-joining (NJ) method with default setting in Clustal X2, and examined 1000 bootstrap replicates. For maximum likelihood (ML) and Bayesian inference (BI) analyses, the best nucleotide substitution model for the data set was selected using the Akaike information criterion (AIC) via MEGA X^46^, and the GTR + G + I model was selected. ML analyses were performed via MEGA X using the nearest-neighbor interchange (NNI) branch-swapping algorithm and 1000 bootstrap replicates to estimate node support values. BI analyses were conducted using the Markov chain Monte Carlo (MCMC) method implemented in MrBayes v3.2.6^47^. MCMC was run for 10^8^ generations with four chains, and trees were sampled every 1000th generation. The fixed number of samples (250,000) was discarded as burn-in, and convergence was checked by Tracer v1.6^48^.

### Secondary structure prediction of SSU rRNA

SSU rDNA sequences of LxSd1, *Pseudodidymocystis* species, *Volvox carteri* (X53904), and *Heterochlorella luteoviridis* (Chodat) Neustupa, Nemcova, Eliás et Skaloud (X73998, Trebouxiophyceae) were aligned in Clustal X2. The secondary structures of the latter two sequences were published previously^13,26^, and each nucleotide of the former sequences were confirmed based on reference to the helix positions in known structures. The LxSd1 SSU rRNA structure was constructed mainly based on that of *H*. *luteoviridis*, as far as it was concordant with this structure. Helices not shown in *Volvox*^26^, that is those between helices 23 and 24 (see, Fig. 6), were constructed with reference to those of *Micractinium pusillum* Fresenius^29^ (Trebouxiophyceae) and distantly related euglenophytes^28^. The helix numbers were labelled according to eukaryotic model by Wuyts *et al*.^27^. Tips of helix structures were also thermodynamically predicted (in silico) via Mfold^49,50^ (http://mfold.rna.albany.edu/?q=mfold).

### Secondary structure prediction and sequence comparison of ITS

The ITS2 sequence obtained from LxSd1 was submitted to the ITS2 database III^51^ web server (http://its2-old.bioapps.biozentrum.uni-wuerzburg.de), where the boundaries of 5.8S, ITS2, and LSU rDNA were determined. Each helix was determined using an RNA folding program with default settings (energy minimization folding) in Mfold. Differences between the ITS2 sequences of LxSd1 and *Pseudodidymocystis* species were determined in Clustal X2.

The ITS1 sequences of LxSd1 and *Pseudodidymocystis* species were folded with the help of Mfold. Common helices for them were determined and then ITS1 structure for LxSd1 was build (Suppl. Fig. 5).

## Supplementary information

Supplementary video.

Supplementary figures.
