# Supplementary material for: Pediludiella daitoensis gen. et sp. nov. (Scenedesmaceae, Chlorophyceae), a large coccoid green alga isolated from a Loxodes ciliate
Source: Sci Rep. 2020 Jan 20;10:628. doi: 10.1038/s41598-020-57423-x (PMC6971069; doi:10.1038/s41598-020-57423-x)
Supplement: Supplementary file 2 — Supplementary figures. [file 41598_2020_57423_MOESM2_ESM.pdf]

Supplementary Figures

Pediludiella daitoensis gen. et sp. nov. (Scenedesmaceae, Chlorophyceae), a large coccoid green alga isolated from a *Loxodes* ciliate

Ryo Hoshina, Masashi M Hayakawa, Mayumi Kobayashi, Rina Higuchi, Toshinobu Suzaki

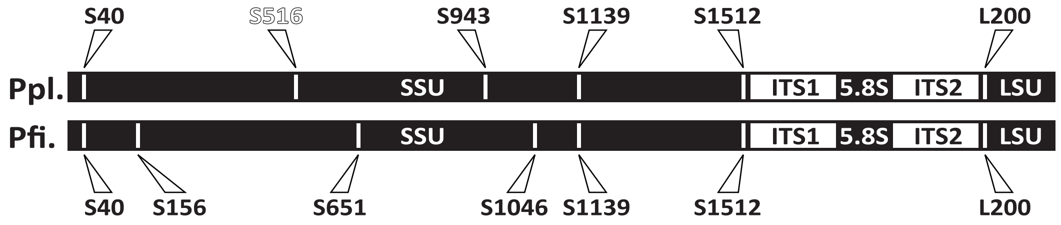

Supplementary Fig. 2. Survey of the ribosomal DNA sequences of *Pseudodidymocystis planctonica* SAG 40.98 (Ppl.) and *P. fina* SAG 2088 (Pfi.) with insertion sites of group I introns. The numbering reflects their homologous positions in the *Escherichia coli* rRNA gene: S, small subunit of rRNA; L, large subunit of rRNA. Ppl.S516 is the only subgroup IE intron, whereas all the other introns are IC1. No introns were found in the LxSd1 sequence.

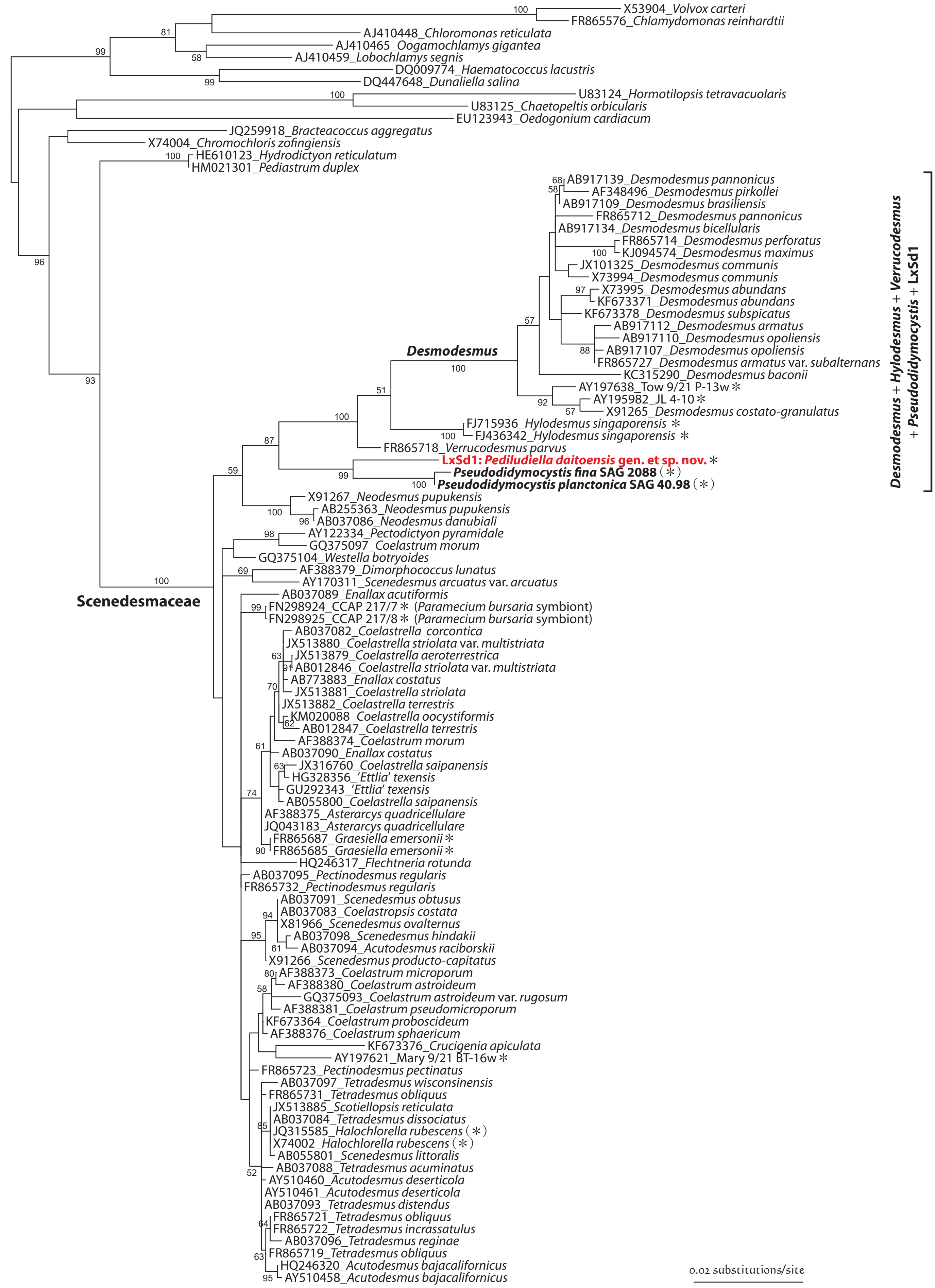

Supplementary Fig. 3. Maximum likelihood tree under the GTR + I + G evolutionary model. Numbers at each node represent bootstrap probabilities; only values above 50% are shown. Algal strains sequenced in this study are shown in bold type. The algae with a spherical shape are shown with asterisks.

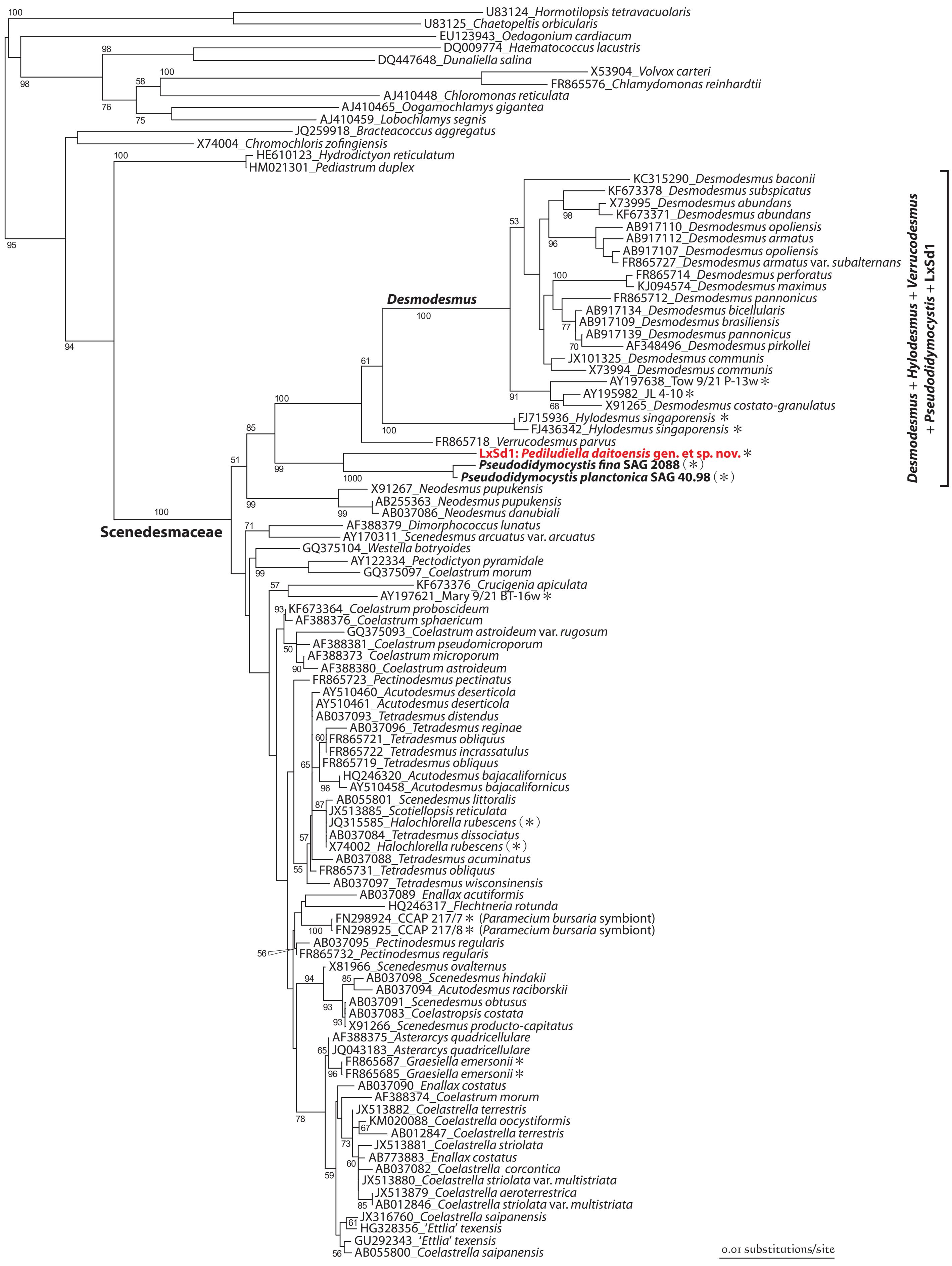

Supplementary Fig. 4. Neighbor-joining tree for Scenedesmaceae based on SSU rDNA sequences. Numbers at each node represent bootstrap probabilities; only values above 50% are shown. Algal strains sequenced in this study are shown in bold type. The algae with a spherical shape are shown with asterisks.

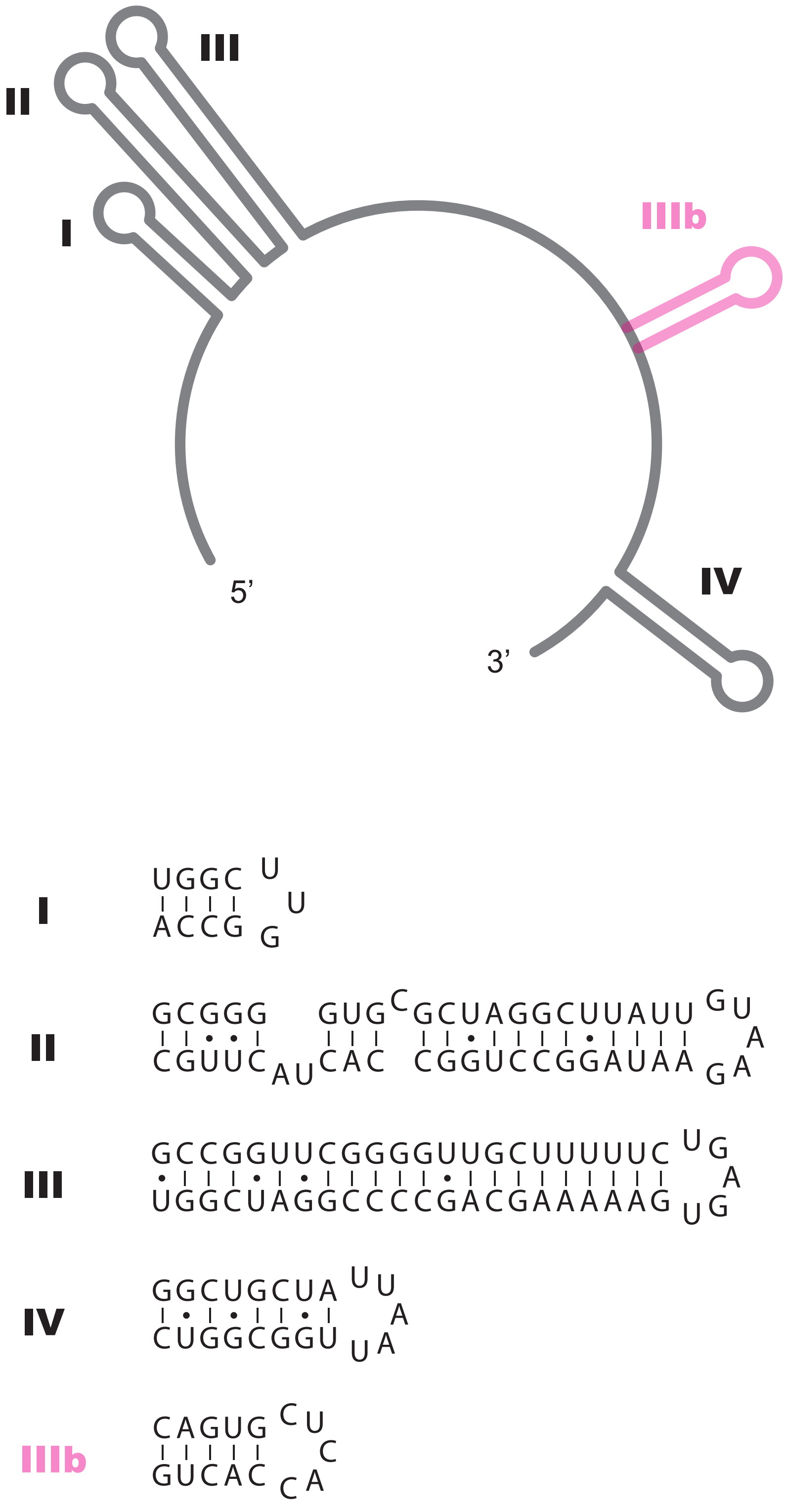

Supplementary Fig. 5. Secondary structure diagram for ITS1 from *Pediludiella daitoensis*. The diagram is folded with the help of Mfold and comparisons with those of *Pseudodidymocystis planctonica* SAG 40.98 and *P. fina* SAG 2088. Large open single-strand with four helices are found as a common structure for them (shown in gray), whereas an additional helix (shown in red) emerges in the predicted model for *P. daitoensis*.
